# Pharmacokinetics, Milk Residues, and Toxicological Evaluation of a Single High Dose of Meloxicam Administered at 30 mg/kg per os to Lactating Dairy Cattle

**DOI:** 10.3390/vetsci10040301

**Published:** 2023-04-19

**Authors:** Scott A. Fritz, Steve M. Ensley, Jay R. Lawrence, Nicholas Van Engen, Zhoumeng Lin, Michael D. Kleinhenz, Larry W. Wulf, Somchai Rice, Patrick J. Gorden, Jackie Peterson, Johann F. Coetzee

**Affiliations:** 1 Department of Anatomy and Physiology, Kansas State University, Manhattan, KS 66506, USA; 2 Department of Veterinary Diagnostic and Production Animal Medicine, Iowa State University, Ames, IA 50011, USA; 3 Department of Environmental and Global Health, University of Florida, Gainesville, FL 32610, USA; 4 Department of Clinical Sciences, Kansas State University, Manhattan, KS 66506, USA

**Keywords:** meloxicam, overdose, cattle, toxicity

## Abstract

**Simple Summary:**

Pain control is a major concern for cattle producers, consumers, and veterinarians. There are currently no analgesic drugs labeled for pain control in lactating dairy cattle in the United States. However, veterinarians are permitted to use analgesic drugs approved in other species in an extra-label manner, but the safety profile of these formulations, especially non-steroidal anti-inflammatory drugs (NSAIDs) administered at high doses, is lacking. NSAID toxicosis is rarely reported in cattle, and the risk level for meloxicam is unknown. This report examined the pharmacokinetics, milk residues, and toxicologic outcomes of a single 30 mg/kg oral dose of meloxicam in lactating dairy cattle.

**Abstract:**

Adverse effects associated with overdose of NSAIDs are rarely reported in cattle, and the risk level is unknown. If high doses of NSAIDs can be safely administered to cattle, this may provide a longer duration of analgesia than using current doses where repeated administration is not practical. Meloxicam was administered to 5 mid-lactation Holstein dairy cows orally at 30 mg/kg, which is 30 times higher than the recommended 1 mg/kg oral dose. Plasma and milk meloxicam concentrations were determined using high-pressure liquid chromatography with mass spectroscopy (HPLC-MS). Pharmacokinetic analysis was performed by using noncompartmental analysis. The geometric mean maximum plasma concentration (C_max_) was 91.06 µg/mL at 19.71 h (T_max_), and the terminal elimination half-life (T_1/2_) was 13.79 h. The geometric mean maximum milk concentration was 33.43 µg/mL at 23.74 h, with a terminal elimination half-life of 12.23 h. A thorough investigation into the potential adverse effects of a meloxicam overdose was performed, with no significant abnormalities reported. The cows were humanely euthanized at 10 d after the treatment, and no gross or histologic lesions were identified. As expected, significantly higher plasma and milk concentrations were attained after the administration of 30 mg/kg meloxicam with similar half-lives to previously published reports. However, no identifiable adverse effects were observed with a drug dose 30 times greater than the industry uses within 10 days of treatment. More research is needed to determine the tissue withdrawal period, safety, and efficacy of meloxicam after a dose of this magnitude in dairy cattle.

## 1. Introduction

Adequate pain management in veterinary medicine is an important consideration when elective invasive procedures are performed or when animals experience pain associated with disease. Veterinarians should be aware of the compounds available for use, their efficacy in abating pain, and any undesirable effects they may have on the animals being treated. In cattle, non-steroidal anti-inflammatory drugs (NSAIDs) are commonly used to treat pain. Meloxicam is an NSAID used in the EU and Canada for alleviating pain associated with disbudding and as adjunctive therapy when treating a variety of infectious diseases [1]. Meloxicam has been shown to be efficacious in improving treatment outcomes in cattle associated with neonatal diarrhea, varying forms of castration, as well as cesarean section surgeries [2,3,4]. Meloxicam is not currently approved for use in food animals in the United States. However, in a recent survey, 16.1% of producers and 80.5% of veterinarian respondents indicated that they had knowledge of and felt comfortable with using oral meloxicam in an extra-label manner in cattle to alleviate pain [5]. 

Meloxicam acts by inhibiting the synthesis of prostaglandin and has anti-inflammatory, anti-exudative, analgesic, and antipyretic activities [6]. Meloxicam is recommended to be administered extra-label to cattle orally at 1 mg/kg of body weight in the US and in accordance with the labeled product in Canada (Meloxicam Oral Suspension USP, Solvet, Calgary, Canada). However, despite widespread familiarity with the extra-label use of meloxicam in the United States, there is no established risk level for adverse effects related to such use in cattle. Moreover, characterizing the margin of safety between efficacy and toxicity could lead to the safe use of higher doses of meloxicam that produce higher plasma drug concentrations for longer and thus provide pain management over an extended period of time after a single dose. Protocols involving higher than currently recommended doses of oral meloxicam could thus advance the welfare of treated animals, especially in situations where repeated therapy is not practical.

The acute oral toxicity of meloxicam has been investigated in other species and ranges from 83.5 mg/kg in rats to >1600 mg/kg in mini-pigs [1]. However, the margin of safety between effectiveness and toxicity has not been well-characterized [7]. There is a growing body of work suggesting that higher and more frequent dosing of NSAIDs may be needed to achieve adequate analgesia in laboratory rodents [7,8,9]. An oral dose of 8 mg/kg was delivered to Wistar rats for 28 continuous days, which resulted in severe ulcerative lesions and hemorrhage in the gastrointestinal tract [10]. However, a single oral dose of 20 mg/kg meloxicam in mice was well tolerated [11]. Furthermore, while meloxicam administered subcutaneously at 20 mg/kg caused injection site lesions, there were no systemic signs of toxicity [9]. Another group used subcutaneous injections of 20 mg/kg meloxicam in mice for 3 or 7 days continuously [8]. Fecal occult blood was associated with treatment; gastritis was not associated with treatment and no other lesions were detected. A subset of the different treatment groups was allowed 7 days to recover, and no differences could be identified from baseline at that time, suggesting the lesions are clinically insignificant and recovery is likely. American kestrels were given oral doses of 20 mg/kg meloxicam every 12 h for 7 days with no evidence of toxicity [12]. The use of NSAIDs at calving has been associated with retained fetal membranes, yet other studies have been unable to detect a difference between NSAID-treated cows and non-NSAID-treated cows [13,14,15]. Effects on reproduction may be as economically significant as individual toxicosis. Taken together, multiple high doses of meloxicam should be used with caution, but a single high dose of meloxicam appears to be well tolerated in multiple species and could result in a longer duration of analgesia without the need for repeated dosing.

Sows were administered 30 mg/kg meloxicam per os for 3 consecutive days in an attempt to deliver adequate meloxicam for analgesia in milk to their offspring [16]. Minor lesions in the gastrointestinal tract were noted at necropsy in some animals, but this dose was well-tolerated clinically. The sow study serves as the basis for the selected dose in the present study of 30 mg/kg per os due to its lack of clinical toxicity even when administered for three consecutive days. The adverse effects of NSAID overdoses in other species have been reported, and ruminants are considered to be at risk for similar adverse effects [17]. The present report investigated the pharmacokinetics, milk residue depletion characteristics, and toxicologic response to a single, large oral dose approximately 30 times the recommended dose used for therapy in lactating cows. If a single high dose of meloxicam in place of repeated dosing is well tolerated in cattle, a longer duration of analgesia may be achievable.

## 2. Materials and Methods

This protocol was approved by the Institutional Animal Care and Use Committee (IACUC) at Iowa State University (ISU), protocol 5-12-7363B. The study design is graphically represented in Table 1.

### 2.1. Experimental Animals

Five clinically healthy adult Holstein–Friesian cows weighing between 600–800 kg were acquired from the ISU Dairy Farm. All animals were currently in lactation, producing between 12.5–21.5 kg of milk per day. Prior to the study, each cow’s health was screened utilizing a physical exam, serum chemistry, and complete blood count.

### 2.2. Housing and Husbandry

Phase I was conducted in the ISU Lloyd Veterinary Medical Center with a single animal. The cow was housed in the large animal medicine facilities in a 12.6 m^2^ individual stall bedded with rubber mats and pine wood shavings. After the successful completion of phase I, the four remaining animals were housed at the ISU Dairy Farm in individual 28 m^2^ pens bedded with sand and straw. For the duration of the study, all animals were maintained on a total mixed ration consisting of corn silage, hay, cotton, fine ground corn, corn gluten, soy plus, soybean meal, and grain mix. This ration was composed of 16.4% crude protein and provided a net energy of 35.72 megacalories per kilogram. Water and feed were offered ad libitum.

### 2.3. Meloxicam Administration

A single oral dose of meloxicam (Mylan Pharmaceuticals Inc., Morgantown, WV, USA; NDC 0378-1089-01; Lot 3032625) was administered to each cow at time zero. The 30 mg/kg dose was administered via balling gun in the form of 1-ounce gelatin capsules (Torpac, Inc., Fairfield, NJ, USA) containing 15 mg meloxicam tablets. Individual doses ranged from 29.98 to 30.02 mg/kg as tablets were left whole to preserve the integrity of the drug.

### 2.4. Sample Collection

Intravenous jugular catheters (Abbocath-T, 14 G × 140 mm) were aseptically placed in each animal and sutured into the skin using 3-0 nylon suture (Ethicon, Inc., Raritan, NJ, USA) 16 h prior to the study’s start. Throughout the study, catheter patency was maintained using a heparin saline flush containing 2 USP units of heparin sodium/mL saline (Heparin Sodium Injection, Baxter Healthcare, Deerfield, IL, USA). Blood samples were collected once daily for complete blood counts and serum chemistry analysis. Approximately 15 mL of whole blood was drawn from the indwelling jugular catheter and immediately transferred into both an 8.5 mL serum separator vacutainer tube (BD Diagnostics, Franklin Lakes, NJ, USA) and a 5 mL vacutainer tube containing 15% EDTA (BD Diagnostics). The vacutainer tubes were refrigerated until they were submitted for analysis. Samples were submitted to the ISU Clinical Pathology Laboratory for serum chemistry analysis and complete blood counts with manual differential. Blood samples for plasma drug analysis were collected at each time point daily (05:00, 10:00, 15:00, 19:00, 24:00). Whole blood was drawn from the jugular catheter and placed into a 10 mL lithium heparin vacutainer tube (BD Diagnostics, Franklin Lakes, NJ, USA). The samples were immediately centrifuged for 10 min at 1600× *g*. Plasma was then pipetted to 2 mL cryovials (Fisher Scientific International, Inc., Hanover Park, IL, USA) and frozen at −70 °C prior to analysis. 

Physiological monitoring included daily temperature, heart rate, respiratory rate, urinalysis, and fecal occult blood test. Vital signs (heart rate, respiratory rate, temperature) were assessed daily. Urine was collected in a polycarbonate vial (Fischer Scientific, Hampton, NH, USA) once daily via free catch. Urine analyses were performed immediately following the free catch and consisted of a specific gravity via refractometer and a urine reagent dipstick analysis (Multistix 10 SG, Siemens Healthcare Diagnostics, Inc., Tarrytown, NY, USA). Assessment of fecal occult blood was performed daily using a fresh stool sample and a Seracult (Propper Manufacturing Co, Inc., Long Island City, NY, USA) rapid card test. 

**Figure 1:**
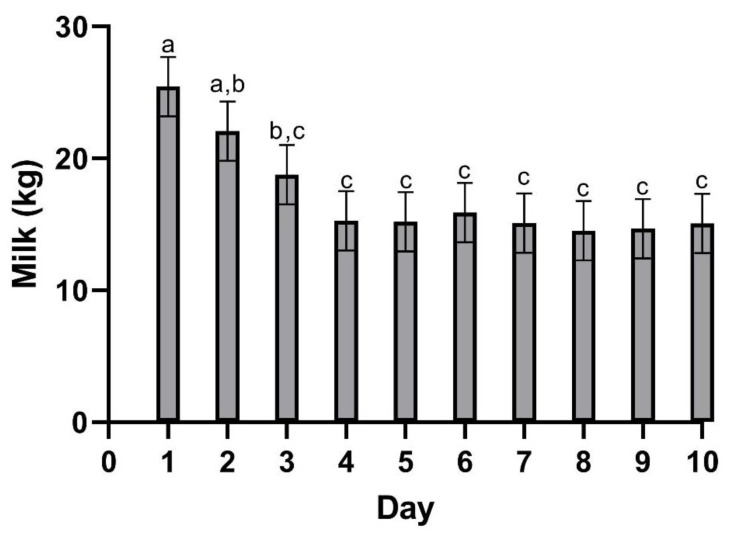
Mean (±SEM) milk production following a single oral dose of meloxicam at 30 mg/kg. ^a,b,c^ Different letters above data bars are statistically significant (*p* = 0.001).

Milk collection for milk drug analysis was performed at each time point (05:00, 10:00, 15:00, 19:00, 24:00) for the duration of the study. Prior to collection, each teat was pre-dipped using a hydrogen peroxide-based dip and fore-stripped to check for signs of mastitis. Milk weights are presented in Figure 1. Following the milking procedure, a 45 mL sample was then taken from the collection vessel and placed in a polycarbonate vial (Fisher Scientific). Samples were immediately taken to the laboratory and frozen at −70 °C prior to analysis. After each collection, the teats were treated with iodine-based post-milking dip. All residual milk collected during the study was discarded due to potential drug residues. 

At the end of the study, 10 days after treatment, all cows were humanely euthanized via captive bold and exsanguination, and necropsy was performed by a trained diagnostician (SE). Gross lesions were noted, and histopathologic evaluation was performed on selected tissues expected to be impacted by NSAID toxicity.

### 2.5. Milk Sample Preparation

After thawing at room temperature, milk samples, milk spikes, and milk QC samples (1.0 mL in 16 × 150 mm glass screw-top tubes) were fortified with the internal standard piroxicam (10 mL of 10 ng/mL). An equivalent volume of ultra-pure water was added to each sample and vortexed. Then, 0.2 M Disodium EDTA (0.5 mL) was added to each sample and vortexed. Finally, 0.5% acetic acid in acetonitrile (5 mL) was added to each sample and vortexed. Additionally, 0.5 g ± 0.1 g of sodium sulfate fractions were premeasured and added to each sample simultaneously to vigorous vortexing to avoid clumping during salt-out. Tubes were capped tightly and mixed by inversion for 5 min at 300 rpm, then centrifuged at 2000× *g* for 10 min. The top organic layer was transferred to a clean 16 × 100 mm glass tube and was evaporated under a stream of nitrogen at 48 °C to approximately 0.5 mL total volume. One mL of 0.1 ammonium acetate buffer, pH 4.5, was added to each reduced sample.

A solid phase extraction (SPE) clean-up followed using a positive pressure manifold (UCT, Inc., Bristol, PA, USA). Strata-X SPE cartridges (60 mg/3 mL, Phenomenex, Torrance, CA, USA) were conditioned and equilibrated sequentially with methanol (2 mL), ultra-pure water (2 mL), then 0.1 M ammonium acetate buffer, pH 4.5 (1 mL). The buffered samples were loaded onto the cartridges under gravitational flow. The cartridges were washed with 5% acetonitrile in 1% formic acid (0.5 mL) and dried under full vacuum for 5 min. A hexane wash (0.5 mL) followed, with an additional 5-min dry under full vacuum. Target analytes were eluted with 2 × 1 mL fractions of 70:30 acetonitrile to methanol. Eluent was reduced to dryness under a stream of nitrogen at 48 °C. 150 µL of 25% acetonitrile in water was added to reconstitute the sample residues and vortexed briefly. Then, 50 µL of ultra-pure water was added and vortexed briefly. The entire reconstituted samples were transferred to labelled auto-sampler vials equipped with glass inserts for analysis by LC-MS.

### 2.6. Plasma Sample Preparation

After thawing at room temperature, plasma samples, plasma spikes, and plasma QC samples (0.2 mL) were treated with 30% perchloric acid (20 mL) after the addition of the internal standard piroxicam (10 mL of 10 ng/mL). The samples were vortexed for 5 s and centrifuged for 20 min at 2000× *g* to sediment the precipitate. A portion of supernatant, 100 μL, was transferred to an injection vial fitted with a glass insert containing 100 μL of 1.5% ammonium hydroxide in 25% aqueous acetonitrile for analysis by LC-MS.

### 2.7. Determination of Meloxicam Concentrations by LC-MS

Plasma and milk concentrations of meloxicam were determined using high-pressure liquid chromatography (Surveyor MS Pump and Autosampler, Thermo Scientific, San Jose, CA, USA)-tandem mass spectrometry (TSQ Quantum Discovery MAX, Thermo Scientific, San Jose, CA, USA). The sample injection volume was set to 12.5 μL. The mobile phases consisted of A: 0.1% formic acid in water and B: 0.1% formic acid in acetonitrile at a flow rate of 0.280 mL/min. The mobile phase began at 40% B with a linear gradient to 95% B at 4 min, which was maintained for 1.5 min, followed by re-equilibration to 40% B. Separation was achieved with a solid-core C18 column (KinetexXB -C18, 100 mm × 2.1 mm, 2.6 µm particles, Phenomenex, Torrance, CA, USA) maintained at 40 °C. Piroxicam eluted at 2.96 min and meloxicam at 4.31 min. Four SRM transitions were monitored for meloxicam, and three SRM transitions were used with the internal standard, piroxicam. The quantifying ions for meloxicam were 72.95, 87.94, 114.91, and 140.89 *m*/*z*, and 77.97, 94.98, and 120.98 *m*/*z* for piroxicam. Sequences consisting of milk and plasma blanks, calibration spikes, QC samples, and bovine milk and plasma samples were batch processed with a processing method developed in the Xcalibur software (Thermo Scientific, San Jose, CA, USA). The processing method automatically identified and integrated each peak in each sample and calculated the calibration curve based on a weighted (1/X) linear fit. Plasma concentrations of meloxicam in unknown samples were calculated by the Xcalibur software based on the calibration curve. Results were then viewed in the Quan Browser portion of the Xcalibur software. Twelve calibration spikes were prepared in bovine milk and plasma, covering the concentration range of 5 to 20,000 ng/mL. QC samples were prepared at concentrations of 15, 150, and 1500 ng/mL. QC samples were prepared in duplicate with each set of samples. Calibration curves exhibited a correlation coefficient (r^2^) exceeding 0.998 across the entire concentration range. The QC samples were accepted as passing within 15% of their nominal values.

### 2.8. Noncompartmental Pharmacokinetic Analysis

The plasma and milk time-concentration data were analyzed with a noncompartmental method using a commercial software Phoenix^®^ (Version 8.3, Certara, Inc., Princeton, NJ, USA). The following pharmacokinetic parameters were calculated, including AUC_0-last_ (area under the curve from the time of dosing to the last measurable concentration), AUC_0-∞_ (AUC from dosing time extrapolated to infinity based on the last observed concentration), AUC_Extrap_ (percentage of AUC_0-∞_ due to extrapolation from T_last_ to infinity), AUMC_0-∞_ (area under the first moment curve extrapolated to infinity based on the last observed concentration), C_max_ (maximum observed concentration occurring at T_max_), CL/F (total body clearance per fraction of dose absorbed), λ_z_-HL (terminal elimination half-life), λ_z_ (terminal elimination rate constant, calculated using log-linear regression of the terminal portions of the concentration-versus-time curves), MRT_0-∞_ (mean residence time extrapolated to infinity), T_max_ (time of maximum observed concentration), and Vz/F (volume of distribution based on the terminal phase per fraction of dose absorbed). 

### 2.9. Statistical Analysis

Hematological and serum biochemistry outcomes were entered into spreadsheets prior to statistical analysis (Excel, Microsoft Corporation, Redmond, WA, USA). Data distributions were visually examined to confirm that data were normally distributed. Changes in each of the hematology and serum chemistry variables over time were assessed using One-way Analysis of Variance (ANOVA) and a commercially available statistical software package (JMP ^®^ Pro 16.1.0, SAS Institute, Cary, NC, USA). Means and standard errors were extracted from the statistical reports and tabulated. Statistical significance was designated a priori as *p* < 0.05. Where a significant effect of time on the outcome was reported, the effect was clarified graphically in Excel. 

## 3. Results

### 3.1. Milk

**Figure 2:**
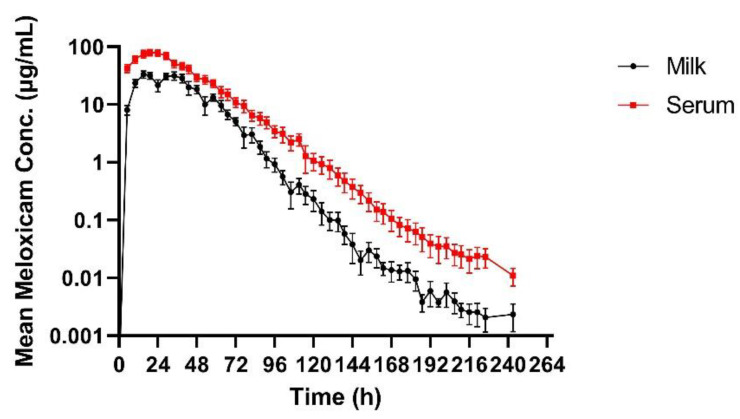
Mean (±SEM) milk and plasma concentrations of meloxicam following a single oral dose of 30 mg/kg in adult Holstein cows (*n* = 5).

**Figure 3:**
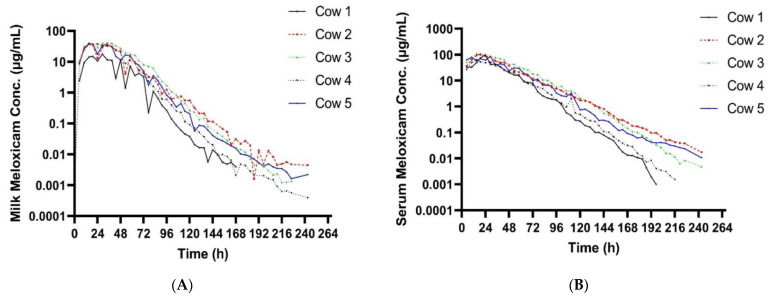
(**A**) Individual milk meloxicam concentrations for 5 cows administered meloxicam at 30 mg/kg PO at time zero. (**B**) Individual plasma meloxicam concentrations for 5 cows administered meloxicam at 30 mg/kg PO at time zero.

Table 2 summarizes the pharmacokinetic parameters of meloxicam in milk. The geometric mean peak milk concentration was 33.43 (17.70–41.39) µg/mL. This occurred at 23.74 (15–34) h. The geometric mean terminal elimination half-life in milk was 12.23 (10.59–14.4) h, and the geometric mean AUC_0-inf_ was 1360.77 h × µg/mL. Mean daily milk production is displayed in Figure 1—there is a slight decrease in milk production over the course of the study. Mean (±SEM) plasma and milk concentrations of meloxicam are presented in Figure 2. Individual milk concentrations of meloxicam are presented in Figure 3A.

### 3.2. Plasma

Summary plasma pharmacokinetic parameters for meloxicam are displayed in Table 3. The geometric mean peak plasma concentration was 91.06 (63.13–110.58) µg/mL at 19.71 (15–29) h. The geometric mean terminal elimination half-life was 13.79 (11.33–17.42) h, oral clearance was 9.09 mL/h/kg, and the bioavailability was 180.77 mL/kg. Individual plasma concentrations of meloxicam are presented in Figure 3B. 

### 3.3. Complete Blood Counts and Blood Chemistry

**Figure 4:**
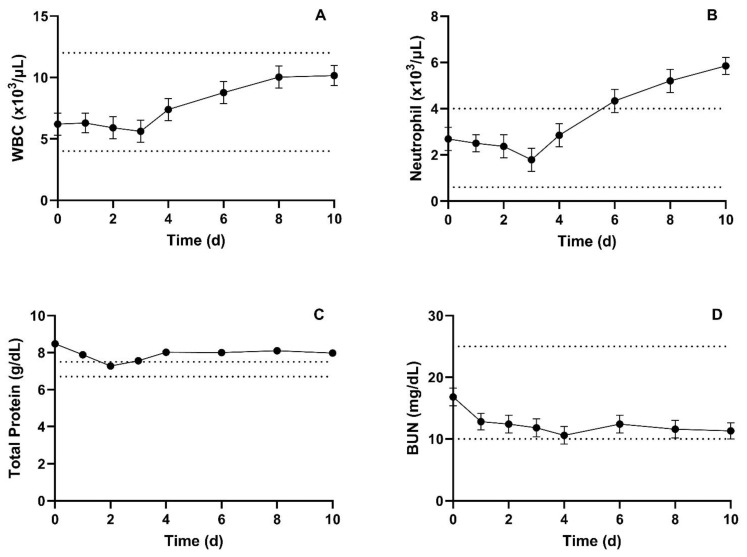
Summary mean (±SEM) WBC counts (**A**), Neutrophil counts (**B**), total protein (**C**), and BUN (**D**) prior to, and for 10 days following, administration of 30 mg/kg meloxicam. Dotted lines denote provided upper and lower reference limits.

Summary of mean (±SEM) CBC parameters are presented in Table 4. CBC parameters that showed significant changes include WBCs, MCHC, Neutrophils, basophils, and plasma protein. WBC counts and neutrophils increased throughout the study, with neutrophils being above the reference ranges for the last three measures. Mean WBC and neutrophil data are presented in Figure 4A,B respectively. WBC counts were within the normal range for the entirety of the study. Hematocrit and red blood cell analyses were normal across all individuals for the entirety of the study. Plasma protein had significant day-to-day variation and was above the reported reference range at most time points, including baseline. 

Summary of mean (±SEM) serum blood chemistry parameters are presented in Table 5. Serum blood chemistry parameters that showed significant differences include potassium, total protein, albumin, total bilirubin, and anion gap. While potassium, albumin, and total bilirubin had significant day-to-day variation, the measured values were still within the reported normal ranges at all time points except for potassium 2 days post-administration. Total protein had significant day-to-day variation and was above the reference range at all but one time point. However, the baseline measure was numerically higher than all other measures, and there was a slight return towards the normal range over time. Mean total protein and BUN are presented in Figure 4C,D, respectively. The anion gap was significantly lower post-administration compared to baseline.

### 3.4. Fecal Occult Blood

Fecal occult blood analyses were negative for all individuals at all time points. 

### 3.5. Urine Analysis

Individual cow urine analyses are presented in Table 6. Urine-specific gravity was evaluated daily and remained normal for four animals at all time points; the fifth animal had lower USG at the time of baseline measurement and throughout the study.

### 3.6. Necropsy Findings

Gross lesions included mild, non-suppurative superficial abomasitis in 4 of 5 cows and were considered mild and incidental findings by the pathologist, along with notable phlebitis around the indwelling catheter in one animal.

### 3.7. Histological Examination

Gross and histologic examination of the kidneys on all animals revealed no evidence of nephritis. NSAID-induced changes in the liver can be both cholestatic and parenchymal in nature [6]. Gross and microscopic evaluation of all livers was unremarkable. Histologically, no lesions were appreciated in the gastrointestinal tract or liver in any of the five animals. One cow had minute foci of lymphoplasmacytic inflammatory aggregates in the renal cortical and medullary interstitium.

## 4. Discussion

Milk and plasma pharmacokinetic parameters reported in this study were different when compared to other published data. The C_max_ in the present study was similar to sows dosed with the same dose (30 mg/kg meloxicam PO) approaching 100 ug/mL [18]. Plasma C_max_ was approximately 30 times higher than the reported C_max_ for studies using 1 mg/kg dose in cattle that reported plasma C_max_ ranging from 1.45–3.10 µg/mL [19,20,21,22,23]. The much higher C_max_ and longer T_max_ were likely directly due to the delivered dose being 30 times higher. The T_max_ in these studies in cattle were shorter than the present study ranging from 10.48–16.75 h compared to 19.7 h. The terminal elimination half-life presented here was similar to other data in cattle using a much smaller dose. This also suggests that excretion is not impacted by the increased dose. Oral clearance was also similar to cattle studies using a 1 mg/kg PO dosing regimen [19,22]. Plasma concentrations following 30 mg/kg meloxicam administrations at 120th hour were similar to the Cmax reported in 1 mg/kg dose studies in cattle. This suggests that a 30 mg/kg oral dose could provide prolonged plasma concentrations and possibly prolonged analgesia. The terminal elimination half-life in the present study in cows was shorter than the reported half-lives in calves [21]. Multiple reports using various NSAIDs have shown decreased clearance in young animals compared to older animals [24,25,26]. The maximum milk concentration and time to maximum concentration in the present study are much higher than data reported from 1 mg/kg oral dose in cattle [19,20]. In those studies, C_max_ in milk ranged from 0.388–0.421 µg/mL compared to the present study ranging from 17.7–41.39 ug/mL, T_max_ in milk was 9.33 h in one study in cattle, and, while not specified in the text, 8–16 h in the other compared to a milk T_max_ of nearly 24 h in the present study. The mean milk terminal elimination half-life in the present study was 12.23 h compared to the reported milk terminal elimination half-life of 10.38 h in cows receiving 1 mg/kg per os [20]. The data presented here are insufficient to establish a withdrawal period for a single extreme overdose because the plasma and milk concentrations were never below the detection limit of the assay. In the event that extreme doses of meloxicam are accidentally administered in the field, more work is needed to identify the post-treatment interval required to clear meloxicam from the plasma and milk. Previous research has shown significant differences in meloxicam depletion in post-partum and mid-lactation cows [19]. The data suggest that post-partum cows will retain violative drug residues longer than mid-lactation cows, similar to the ones used in our study. In the present study, the slight reduction in milk yield over time is most likely due to frequent handling. 

Inhibition of prostaglandin production is the basis for both the adverse and therapeutic effects of NSAIDs. The most common target organs for NSAID-induced lesions are the gastrointestinal tract, kidney, and liver [27]. Expected lesions in the gastrointestinal tract are irritation, erosion, and ulceration [17]. These effects can lead to anemia via blood loss, protein-losing enteropathy, and melena. NSAID nephropathy in other species is characterized by oliguria, azotemia, hyperkalemia, and nephritis [28]. Thus, these biochemical changes were evaluated to identify any abnormalities related to the toxic potential of a single large oral dose in cattle. BUN and creatinine remained within the reference ranges at all time points. A slight hypokalemia was identified on day 2, and nephritis was not identified in the post-mortem exam or histopathology. 

The toxicologic evaluation of a single oral dose of 30 mg/kg meloxicam was largely unremarkable. Expected target organ function was thoroughly evaluated ante-mortem, and no significant abnormalities were identified. Approximate oral LD50 dosages have been reported in a number of laboratory species ranging from 83.5 mg/kg to >1600 mg/kg, with a maximum non-lethal oral dose in rats of 50 mg/kg [29]. Data evaluating the toxic potential of using large doses in cattle are lacking. Multiple dosing regimens have been used to evaluate the toxic effects of high doses of meloxicam in laboratory animals, with most utilizing daily doses over time. There is one report of a dog accidentally receiving 1 mg/kg IV (intravenously) meloxicam where therapeutic plasma exchange was successfully employed to circumvent any toxicologic effects, so it is unknown if this dose would have resulted in any adverse effects in a dog [30]. Severe clinical signs and lesions occurred in a study using Wistar rats where the rats were orally gavaged with 8 mg/kg meloxicam for 28 days. In the rat study, gross lesions included hemorrhage and congestion of the GI tract, liver, and kidney. These organs also showed histopathologic changes, including vacuolar degeneration of hepatocytes with hemorrhage, hyperplasia of intestinal villi projecting into the lumen, sloughing of the glandular epithelium of the stomach and gastric erosions, as well as tubular degeneration and hemorrhage in the kidneys. Blood parameters were also evaluated in the rat study, with the most relevant changes related to the hemorrhage appreciated on a gross exam (decreases in packed cell volume, total erythrocyte count, and hemoglobin) [10]. A single oral dose of 20 mg/kg meloxicam did not result in any toxicologic effects in one study in mice [11], while a separate study in mice receiving 20 mg/kg meloxicam administered subcutaneously resulted in concentration-dependent lesions at the injection site with no evidence of gastric ulceration or kidney or liver lesions [9]. Meloxicam was administered at 20 mg/kg PO to American Kestrels every 12 h for 7 days with no clinical signs or abnormalities appreciated in PCV and biochemistry, and no evidence of renal toxicity was identified [12]. Llamas were administered 1 mg/kg meloxicam PO once, and no adverse effects were observed [31]. Sows were administered 30 mg/kg meloxicam orally for 3 days to evaluate whether analgesic concentrations could be delivered to their offspring via milk. Button ulcers and gastritis were identified in 2/5 sows and their litters [16]. 

Blood parameters were evaluated in the llama study, and the most significant change was an increase in mean BUN; however, pre- and post-treatment values were still within the utilized reference range and deemed not clinically relevant [31]. CBC changes were reported in Wistar rats given 8 mg/kg meloxicam per os for 28 days, and the abnormalities were directly related to the post-mortem findings of hemorrhage in multiple tissues [10]. The neutrophil count in the present study was the only parameter with a statistical difference from baseline to the end of the study and with values outside of expected reference ranges. The elevation in neutrophil count correlated clinically with the confirmed phlebitis. Post-mortem evaluations were also unremarkable from gross observations to the lack of histologic lesions. The inflammatory lesions in the kidney of one cow were categorized as mild and incidental by the pathologist. The post-mortem findings are similar to multiple reports utilizing a relatively comparable dose for a short time period in other species, suggesting that multiple doses over time are needed to cause appreciable changes.

## 5. Conclusions

Administration of meloxicam at 30 mg/kg per os did not result in any significant, clinically relevant changes that would be considered indicative of NSAIDs toxicity in five lactating dairy cattle within 10 days of treatment. These results suggest a single large oral dose is well-tolerated by lactating dairy cows. This work also suggests a wide safety margin between analgesic effectiveness and clinical toxicity of a single dose of meloxicam. Further work is needed to evaluate a dose of this magnitude in other age classes and multiple doses of this magnitude. Additionally, further work is needed to determine the toxic dose of meloxicam in cattle and the dose, if any, that results in negative impacts on reproduction.

## Figures and Tables

**Table 1:** Graphical representation of the study design calendar.

Study Day	−1	0	1	2	3	4	5	6	7	8	9	10
Catheter	X											
Drug administration		X										
CBC/Chem	X	X	X	X	X	X	X	X	X	X	X	X
Plasma Drug		X	X	X	X	X	X	X	X	X	X	X
Milk Drug		X	X	X	X	X	X	X	X	X	X	X
Physical Exam	X	X	X	X	X	X	X	X	X	X	X	X
Urinalysis	X	X	X	X	X	X	X	X	X	X	X	X
Fecal blood	X	X	X	X	X	X	X	X	X	X	X	X
Euthanasia												X

**Table 2:** Geometric mean milk pharmacokinetic parameters of meloxicam following single oral administration at the dose rate of 30 mg/kg in lactating dairy cows (*n* = 5).

Parameter	Unit	Mean	SD	Median	Range
λ_z_	1/h	0.060	1.130	0.060	0.05–0.07
λ_z_-HL	h	12.230	1.130	11.600	10.59–14.40
AUC_0-last_	h × µg/mL	1360.71	1.580	1553.23	621.102–2035.769
AUC_0-∞_	h × µg/mL	1360.77	1.580	1553.28	621.162–2035.791
AUC_extrap_	%	0.002	3.590	0.003	0.0004–0.01
C_max_	µg/mL	33.4	1.43	39.0	17.704–41.393
T_max_	h	23.740	1.530	29.000	15–34

**Table 3:** Geometric mean plasma pharmacokinetic parameters of meloxicam following single oral administration at 30 mg/kg in lactating dairy cows (*n* = 5).

Parameter	Unit	Mean	SD	Median	Range
λ_z_	1/h	0.050	1.204	0.052	0.04–0.061
λ_z_-HL	h	13.79	1.20	13.41	11.328–17.422
T_max_	h	19.71	1.34	19.00	15–29
C_max_	µg/mL	91.061	1.248	98.164	63.10–110.578
CL/F	mL/h/kg	9.09	1.3	8.5	6.283–12.716
AUC_0–∞_ AUClast	h × µg/mL h × µg/mL	3301.30 3301.16	1.342 1.342	3509.40 3509.16	2359.279–4775.071 2359.278–4774.979
AUC extrap	%	0.001	8.646	0.002	0–0.011
AUMC_0–∞_	h^2^ × µg/mL	109,842.0	1.4	117,505	75,845–172,426
MRT_0–∞_	h	33.27	1.07	33.48	30.515–36.11
Vz/F	ml/kg	181	1	194	121.542–217.102

**Table 4:** Summary of mean (±SEM) CBC parameters prior to meloxicam treatment and for 10 days post-treatment for cows administered 30 mg/kg meloxicam per os (*n* = 5).

Parameter	Days after Meloxicam Administration at 30 mg/kg PO								
(Ref Range)	Baseline	1	2	3	4	6	8	10	*p*-Value
WBC × 10^3^/uL (4–12)	6.20 ^c^ (0.90)	6.29 ^c^ (0.81)	5.9 ^c^ (0.89)	5.62 ^c^ (0.89)	7.39 ^bc^ (0.89)	8.77 ^ab^ (0.89)	10.03 ^a^ (0.89)	10.16 ^a^ (0.90)	<0.001
RBC × 10^6^/uL (5–10)	6.40 ^a^ (0.25)	5.57 ^b^ (0.24)	5.34 ^b^ (0.25)	5.48 ^b^ (0.25)	5.66 ^b^ (0.25)	5.55 ^b^ (0.25)	5.6 ^b^ (0.25)	5.49 ^b^ (0.24)	0.26
Hemoglobin g/dL (5–15)	11.98 ^a^ (0.61)	9.97 ^b^ (0.58)	9.66 ^b^ (0.61)	9.8 ^b^ (0.61)	10.2 ^b^ (0.61)	10.02 ^b^ (0.61)	10.24 ^b^ (0.61)	9.73 ^b^ (0.58)	0.30
Hematocrit % (24–46)	32.9 ^a^ (1.64)	27.33 ^b^ (1.57)	26.34 ^b^ (1.64)	27.12 ^b^ (1.64)	27.88 ^b^ (1.64)	27.40 ^b^ (1.64)	27.64 ^b^ (1.64)	27.01 ^b^ (1.57)	0.29
MCV fl (40–60)	51.20 ^a^ (1.62)	49.04 ^a^ (1.56)	49.26 ^a^ (1.62)	49.36 ^a^ (1.62)	49.16 ^a^ (1.62)	49.3 ^a^ (1.62)	49.28 ^a^ (1.62)	49.26 ^a^ (1.56)	0.99
MCH pg (11–17)	* 18.60 ^a^ (0.61)	* 17.91 ^a^ (0.58)	* 18.06 ^a^ (0.61)	* 17.88 ^a^ (0.61)	* 18.00 ^a^ (0.61)	* 18.02 ^a^ (0.61)	* 18.26 ^a^ (0.61)	* 17.75 ^a^ (0.58)	0.99
MCHC g/dL (30–36)	* 36.38 ^ab^ (0.28)	* 36.56 ^ab^ (0.23)	* 36.68 ^ab^ (0.28)	* 36.22 ^ab^ (0.28)	* 36.58 ^ab^ (0.28)	* 36.58 ^ab^ (0.28)	* 37.04 ^a^ (0.28)	* 36.02 ^b^ (0.23)	0.04
Platelets × 10^3^/uL (100–800)	472.4 ^ab^ (44.34)	450.9 ^ab^ (41.99)	419.6 ^b^ (44.34)	435.8 ^ab^ (44.34)	492.8 ^ab^ (44.34)	452.2 ^ab^ (44.34)	494.4 ^ab^ (44.34)	511.4 ^a^ (41.99)	0.78
Neutrophils × 10^3^/uL (0.6–4.0)	2.69 ^bc^ (0.50)	2.5 ^bc^ (0.37)	2.37 ^bc^ (0.50)	1.79 ^c^ (0.50)	2.85 ^bc^ (0.50)	* 4.34 ^ab^ (0.50)	* 5.20 ^a^ (0.50)	* 5.85 ^a^ (0.37)	<0.001
Lymphocytes × 10^3^/uL (2.5–7.5)	2.74 ^b^ (0.75)	3.22 ^ab^ (0.73)	2.81 ^b^ (0.75)	3.12 ^ab^ (0.75)	3.84 ^ab^ (0.75)	3.49 ^ab^ (0.75)	4.00 ^a^ (0.75)	3.49 ^ab^ (0.73)	0.98
Monocytes × 10^3^/uL (0.03–0.85)	0.348 ^a^ (0.07)	0.165 ^a^ (0.05)	0.156 ^a^ (0.07)	0.218 ^a^ (0.07)	0.216 ^a^ (0.07)	0.308 ^a^ (0.07)	0.368 ^a^ (0.07)	0.294 ^a^ (0.05)	0.24
Eosinophils × 10^3^/uL (0.0–2.4)	0.338 ^a^ (0.18)	0.374 ^a^ (0.16)	0.506 ^a^ (0.18)	0.298 ^a^ (0.18)	0.420 ^a^ (0.18)	0.598 ^a^ (0.18)	0.418 ^a^ (0.18)	0.388 ^a^ (0.16)	0.96
Basophils × 10^3^/uL (0.0–0.2)	0.082 ^a^ (0.07)	0.021 ^a^ (0.05)	0.050 ^a^ (0.07)	0.024 ^a^ (0.07)	0.058 ^a^ (0.07)	0.012 ^a^ (0.07)	0.054 ^a^ (0.07)	* 0.221 ^a^ (0.05)	0.02
RDW % (8–15)	* 17.78 ^a^ (0.47)	* 17.63 ^a^ (0.46)	* 17.52 ^a^ (0.47)	* 17.44 ^a^ (0.47)	* 17.34 ^a^ (0.47)	* 17.5 ^a^ (0.47)	* 17.52 ^a^ (0.47)	* 17.46 ^a^ (0.46)	0.99
Plasma Protein g/dL (6.9–7.7)	* 8.76 ^a^ (0.22)	* 8.33 ^abc^ (0.17)	7.64 ^c^ (0.22)	* 7.86 ^bc^ (0.22)	* 8.48 ^abc^ (0.22)	* 8.32 ^abc^ (0.22)	* 8.6 ^ab^ (0.22)	* 8.63 ^ab^ (0.17)	0.02
Fibrinogen mg/dL (100–500)	* 540 ^ab^ (77.82)	490 ^b^ (64.60)	440 ^b^ (77.82)	460 ^b^ (77.82)	* 520 ^ab^ (77.82)	* 560 ^ab^ (77.82)	* 620 ^ab^ (77.82)	* 720 ^a^ (64.60)	0.12

^a,b,c^ Different letters within rows are statistically significant (*p* = 0.05). * Indicates values that are outside the reference range provided by the analyzing laboratory. WBC, white blood cells; RBC, red blood cells; MCV, mean corpuscular volume; MCH, mean cell hemoglobin; MCHC, mean corpuscular hemoglobin concentration; RDW, red cell distribution width.

**Table 5:** Summary of mean (±SEM) serum blood chemistry parameters prior to meloxicam treatment and for 10 days post-treatment for cows administered 30 mg/kg meloxicam per os (*n* = 5).

Parameter	Days after Meloxicam Administration at 30 mg/kg PO								
(Ref. Range)	Baseline	1	2	3	4	6	8	10	*p*-Value
Sodium mEq/L (132–152)	142 ^a^ (0.92)	140 ^a^ (0.75)	140.2 ^a^ (0.92)	139.6 ^a^ (0.92)	141.4 ^a^ (0.92)	140.6 ^a^ (0.92)	140.6 ^a^ (0.92)	140.5 ^a^ (0.75)	0.21
Potassium mEq/L (3.9–5.8)	4.5 ^a^ (0.12)	3.96 ^bc^ (0.09)	* 3.8 ^c^ (0.12)	3.94 ^bc^ (0.12)	4.2 ^abc^ (0.12)	4.22 ^abc^ (0.12)	4.36 ^ab^ (0.12)	4.28 ^ab^ (0.09)	0.01
Chloride mEq/L (100–115)	100.6 ^b^ (1.40)	102.8 ^ab^ (1.22)	105.2 ^a^ (1.40)	103.8 ^ab^ (1.40)	102 ^ab^ (1.40)	102.2 ^ab^ (1.40)	101.2 ^ab^ (1.40)	102.8 ^ab^ (1.22)	0.60
Bicarbonate mEq/L (21–31)	29 ^a^ (1.13)	28.5 ^a^ (0.87)	26.8 ^a^ (1.13)	27.2 ^a^ (1.13)	*31.2 ^a^ (1.13)	30 ^a^ (1.13)	29.4 ^a^ (1.13)	29.5 ^a^ (0.87)	0.18
Calcium mg/dL (8.0–11.4)	9.14 ^ab^ (0.27)	8.98 ^ab^ (0.22)	8.44 ^b^ (0.27)	8.78 ^ab^ (0.27)	9.64 ^a^ (0.27)	9.06 ^ab^ (0.27)	9.4 ^ab^ (0.27)	9.52 ^a^ (0.22)	0.06
Phosphorus mg/dL (5.6–8.0)	6.88 ^a^ (0.68)	7.21 ^a^ (0.47)	7.66 ^a^ (0.68)	6.18 ^a^ (0.68)	6.38 ^a^ (0.68)	6.34 ^a^ (0.68)	5.82 ^a^ (0.68)	5.75 ^a^ (0.47)	0.15
BUN mg/dL (10–25)	16.8 ^a^ (1.45)	12.8 ^b^ (1.33)	12.4 ^b^ (1.45)	11.8 ^b^ (1.45)	10.6 ^b^ (1.45)	12.4 ^b^ (1.45)	11.6 ^b^ (1.45)	11.3 ^b^ (1.33)	0.19
Creatinine mg/dL (0.1–1.8)	1.38 ^a^ (0.14)	1.21 ^b^ (0.13)	1.14 ^b^ (0.14)	1.12 ^b^ (0.14)	1.14 ^b^ (0.14)	1.14 ^b^ (0.14)	1.2 ^ab^ (0.14)	1.16 ^b^ (0.13)	0.97
Glucose md/dL (40–100)	70.4 ^ab^ (3.95)	72.2 ^a^ (3.48)	63.6 ^ab^ (3.95)	65.4 ^ab^ (3.95)	64 ^ab^ (3.95)	62.4 ^ab^ (3.95)	59.6 ^b^ (3.95)	67.6 ^ab^ (3.48)	0.30
Total Protein g/dL (6.7–7.5)	* 8.48 ^a^ (0.16)	* 7.89 ^ab^ (0.12)	7.28 ^c^ (0.16)	* 7.56 ^bc^ (0.16)	* 8.02 ^ab^ (0.16)	* 8.00 ^ab^ (0.16)	* 8.10 ^ab^ (0.16)	* 7.98 ^ab^ (0.12)	0.002
Albumin g/dL (2.5–3.8)	3.38 ^a^ (0.10)	2.93 ^bc^ (0.09)	2.66 ^c^ (0.10)	2.72 ^bc^ (0.10)	2.94 ^bc^ (0.10)	2.9 ^bc^ (0.10)	2.98 ^b^ (0.10)	2.9 ^bc^ (0.09)	0.003
AST U/L (55–125)	98.8 ^a^ (11.74)	95.3 ^a^ (10.53)	76.2 ^a^ (11.74)	83.8 ^a^ (11.74)	90.6 ^a^ (11.74)	84.6 ^a^ (11.74)	82.8 ^a^ (11.74)	74.6 ^a^ (10.53)	0.71
CK U/L (1–350)	265.8 ^a^ (121)	327.7 ^a^ (99)	258.8 ^a^ (121)	248 ^a^ (121)	98 ^a^ (121)	91.6 ^a^ (121)	81.4 ^a^ (121)	64.7 ^a^ (99)	0.55
ALP U/L (25–250)	104.4 ^a^ (16.95)	78 ^ab^ (15.86)	63.8 ^b^ (16.95)	65 ^b^ (16.95)	72 ^ab^ (16.95)	64.6 ^b^ (16.95)	72.8 ^ab^ (16.95)	60.3 ^b^ (15.86)	0.7
GGT U/L (1–50)	* 57.4 ^ab^ (8.59)	* 58.1 ^a^ (8.46)	* 50.6 ^ab^ (8.59)	49.4 ^b^ (8.59)	* 55.8 ^ab^ (8.59)	* 54.4 ^ab^ (8.59)	* 56 ^ab^ (8.59)	* 52.4 ^ab^ (8.46)	0.99
Total Bilirubin mg/dL (0.1–1.6)	0.42 ^a^ (0.04)	0.338 ^ab^ (0.03)	0.146 ^c^ (0.04)	0.186 ^c^ (0.04)	0.222 ^bc^ (0.04)	0.274 ^abc^ (0.04)	0.284 ^abc^ (0.04)	0.291 ^abc^ (0.03)	0.001
Anion Gap (14–21)	17 ^a^ (0.96)	* 12.7 ^b^ (0.71)	* 12.2 ^b^ (0.96)	* 12.6 ^b^ (0.96)	* 12.4 ^b^ (0.96)	* 12.6 ^b^ (0.96)	14.4 ^ab^ (0.96)	* 12.4 ^b^ (0.71)	0.01
Lipemic Indice	20 ^a^ (0)	20 ^a^ (0)	20 ^a^ (0)	20 ^a^ (0)	20 ^a^ (0)	20 ^a^ (0)	20 ^a^ (0)	20 ^a^ (0)	
Hemolytic Indice	32 ^a^ (3.94)	22.3 ^ab^ (2.72)	18.2 ^ab^ (3.94)	15 ^ab^ (3.94)	18.6 ^ab^ (3.94)	15.8 ^ab^ (3.94)	17.8 ^ab^ (3.94)	15.1 ^b^ (2.72)	0.01
Icteric Indice	3.8 ^a^ (0.15)	3.5 ^ab^ (0.12)	2.8 ^c^ (0.15)	2.8 ^c^ (0.15)	3 ^bc^ (0.15)	3 ^bc^ (0.15)	3 ^bc^ (0.15)	3 ^bc^ (0.12)	

^a,b,c^ Different letter within rows are statistically significant (*p* = 0.05). * Indicates values that are outside the reference range provided by the analyzing laboratory. BUN, blood urea nitrogen; AST, aspartate transaminase; CK, creatinine kinase; ALP, alkaline phosphatase; GGT, gamma glutamyltransferase.

**Table 6:** Individual cow urine analysis prior to meloxicam treatment and for 10 days post-treatment for cows administered 30 mg/kg meloxicam per os.

Animal ID	Parameter	Baseline	1	2	3	4	6	8	10
1	Sp. Gravity	1.034	1.04	1.046	1.047	1.042	1.036	1.038	1.032
	pH	8.5	8.5	8.5	8.5	8.5	8.5	8.5	8.5
	Protein	3	3	3	3	1	1	2	2
	Leukocytes	1	1	1	0.5	1	0.5	0.5	1
2	Sp. Gravity	1.013	1.021	N/A	1.023	1.02	1.016	1.015	1.015
	pH	8.5	8.5	8.5	8.5	8.5	8.5	8.5	8.5
	Protein	1	1	0.5	0.5	0.5	0.5	0.5	0.5
	Leukocytes	0	0.5	0.5	0.5	0	0	0	0
3	Sp. Gravity	1.035	1.03	1.044	1.05	1.046	1.031	1.027	1.025
	pH	8.5	8	8.5	8.5	8.5	8.5	8.5	8.5
	Protein	0.5	0.5	1	0.5	1	2	0.5	0.5
	Leukocytes	0.5	0.5	0.5	0.5	0.5	0	0	0
4	Sp. Gravity	1.026	1.045	1.043	1.037	1.036	1.027	1.029	1.028
	pH	8.5	8.5	8.5	8.5	8.5	8.5	8.5	8.5
	Protein	1	1	0.5	3	1	1	1	1
	Leukocytes	1	1	0.5	0.5	0.5	0	0	0
5	Sp. Gravity	1.039	1.03	1.039	1.04	1.03	1.037	1.03	1.027
	pH	8.5	8.5	8.5	8.5	8.5	8.5	8.5	8.5
	Protein	0	0	0	0	0	0	0	0
	Leukocytes	1	0.5	0.5	0.5	0.5	0	0	0

## Data Availability

Data available on request due to privacy concerns relating to live animal use.
